# MousiPLIER: A Mouse Pathway-Level Information Extractor Model

**DOI:** 10.1523/ENEURO.0313-23.2024

**Published:** 2024-06-05

**Authors:** Shuo Zhang, Benjamin J. Heil, Weiguang Mao, Maria Chikina, Casey S. Greene, Elizabeth A. Heller

**Affiliations:** ^1^Department of Systems Pharmacology and Translational Therapeutics, University of Pennsylvania, Philadelphia, Pennsylvania 19104; ^2^Penn Epigenetics Institute, Perelman School of Medicine, University of Pennsylvania, Philadelphia, Pennsylvania 19104; ^3^Genomics and Computational Biology Graduate Group, Perelman School of Medicine, University of Pennsylvania, Philadelphia, Pennsylvania 19104; ^4^Department of Computational and Systems Biology, School of Medicine, University of Pittsburgh, Pittsburgh, Pennsylvania 15260; ^5^Department of Biochemistry and Molecular Genetics, University of Colorado School of Medicine, Denver, Colorado 80045

**Keywords:** aging, gene expression, machine learning

## Abstract

High-throughput gene expression profiling measures individual gene expression across conditions. However, genes are regulated in complex networks, not as individual entities, limiting the interpretability of gene expression data. Machine learning models that incorporate prior biological knowledge are a powerful tool to extract meaningful biology from gene expression data. Pathway-level information extractor (PLIER) is an unsupervised machine learning method that defines biological pathways by leveraging the vast amount of published transcriptomic data. PLIER converts gene expression data into known pathway gene sets, termed latent variables (LVs), to substantially reduce data dimensionality and improve interpretability. In the current study, we trained the first mouse PLIER model on 190,111 mouse brain RNA-sequencing samples, the greatest amount of training data ever used by PLIER. We then validated the mousiPLIER approach in a study of microglia and astrocyte gene expression across mouse brain aging. mousiPLIER identified biological pathways that are significantly associated with aging, including one latent variable (LV41) corresponding to striatal signal. To gain further insight into the genes contained in LV41, we performed *k*-means clustering on the training data to identify studies that respond strongly to LV41. We found that the variable was relevant to striatum and aging across the scientific literature. Finally, we built a Web server (http://mousiplier.greenelab.com/) for users to easily explore the learned latent variables. Taken together, this study defines mousiPLIER as a method to uncover meaningful biological processes in mouse brain transcriptomic studies.

## Significance Statement

RNA-sequencing studies define differential expression of individual genes across conditions. However, genes are regulated in complex networks, not as individual entities. Machine learning models that incorporate biological pathway information are a powerful tool to analyze human gene expression. However, such models are lacking for mouse, despite the vast number of mouse RNA-seq datasets. We trained a mouse pathway-level information extractor model (mousiPLIER) to reduce data dimensionality from over 10,000 genes to 196 “latent variables” that map to known biological pathways. To validate this approach, we applied mousiPLIER to differential expression across mouse brain aging. We identified 26 functional pathways (latent variables) that varied across aging. Finally, we developed a Web server to facilitate use of mousiPLIER by the scientific community.

## Introduction

Over the last decade, scientists have generated an astronomical amount of brain gene expression data (Carulli et al., 1998; Anders and Huber, 2010; Costa-Silva et al., 2017; Keil et al., 2018; Y. Zhang et al., 2021). Differential gene expression analysis of high-throughput RNA-sequencing data is commonly applied to interrogate the relative enrichment of a single transcript across samples. However, genes are regulated in complex networks, rather than as individual entities. Furthermore, gene expression profiling studies are limited in statistical power, as they tend to examine relatively few samples compared with the number of expressed transcripts and increasing the number of samples can be prohibitively expensive.

Machine learning models that incorporate prior pathway information have shown great power in analyzing human gene expression. To this end, we apply an unsupervised learning method that (1) reduces the dimensionality and/or (2) incorporates additional published gene expression datasets. Unsupervised machine learning is a method that defines the structure of “unlabeled data”, for which information on the biological context and experimental conditions is removed. Such methods are well suited for gene expression data and are often used for tasks such as reducing the dimensionality of expression datasets (Hotelling, 1933; der Maaten and Hinton, 2008; McInnes et al., 2018), clustering samples (Oyelade et al., 2016; Chen et al., 2020), or learning shared expression patterns across experiments (Tan et al., 2016; Handl et al., 2019). While unsupervised machine learning models are capable of analyzing large amounts of unlabeled expression data, many of them do not explicitly encode prior biological knowledge to encourage the model to learn biologically meaningful patterns of gene expression over technical ones.

A novel approach, the modeling framework pathway-level information extractor (PLIER; Mao et al., 2019), is built explicitly to work on expression data and uses matrix factorization to incorporate prior biological knowledge in the form of sets of genes corresponding to biological pathways or cell type markers. This approach converts gene expression data into a series of values called “latent variables” (LVs) that correspond to potentially biologically relevant combinations of differentially expressed genes. PLIER learns diverse biological pathways from entire compendia of expression data and can transfer that knowledge to smaller studies, such as MultiPLIER (Taroni et al., 2019). However, PLIER models are largely trained on a single dataset rather than a compendium (Rubenstein et al., 2020; Stogsdill et al., 2022; Z. Zhang et al., 2022), and past MultiPLIER runs have only trained models with up to tens of thousands of samples (Taroni et al., 2019; Banerjee et al., 2020).

To expand the application and utility of PLIER for identifying meaningful biological pathways from gene expression data, we trained a PLIER model on a compendium of mouse gene expression data. In doing so, we trained the first mouse compendium PLIER model (mousiPLIER), on the greatest amount of training data (190,111 samples) ever used by this model. We demonstrated successful optimization of the model training, which generated hypotheses on regulation of mouse brain aging. A further innovation applied *k*-means clustering in the latent variable space to identify the microglia-associated latent variables that corresponded to aging-related changes in the training data. Finally, to maximize widespread usability of mousiPLIER, we built a Web server that allows others to visualize the results and find patterns in the data based on their own latent variables of interest. Going forward, this model and its associated Web server will be a useful tool for better understanding mouse gene expression.

## Materials and Methods

### Data

We began by downloading all the mouse gene expression data in Recount3, along with its corresponding metadata (Wilks et al., 2021). We then removed the single-cell RNA-seq data from the dataset to ensure our data sources were consistent across samples and studies. A total of 190,111 samples from mice of either sex were left for downstream processing. Next, we filtered the expression data, keeping only genes that overlapped between Recount3 and our prior knowledge gene sets. Then, we normalized the expression into TPM (transcripts per million) using gene lengths from the Ensembl BioMart database (Howe et al., 2021). Finally, we *Z*-scored the expression data to ensure a consistent range for the downstream PLIER model.

For our prior-knowledge gene sets, we used cell type marker genes from CellMarker (X. Zhang et al., 2019), pathway gene sets from Reactome (Gillespie et al., 2022), and manually curated brain marker genes from Allen Mouse Brain Atlas (https://mouse.brain-map.org; Lein et al., 2007). We selected cell type marker genes corresponding to all available mouse cell types within the CellMarker database. For mouse biological pathways, we downloaded pathway information from the Reactome database. More specifically, we processed the files “Ensembl2Reactome_All_Levels.txt,” “ReactomePathways.txt,” and “ReactomePathwaysRelation.txt,” selecting only pathways using mouse genes, filtering out all pathways with fewer than five genes present, and keeping only pathways that were leaf nodes on the pathway hierarchy. Because we were interested in mouse brains in particular, we rounded out our set of prior information by manually selecting marker genes for the striatum, midbrain, and cerebral cortex. In total, we used 1,003 prior knowledge pathways when training our model.

### PLIER

The mousiPLIER is built on PLIER, which transforms gene expression data into latent variable space with prior biological pathways incorporated using matrix factorization (Mao et al., 2019). The inputs for PLIER are gene expression matrix (*Y*, genes as row and samples as columns), and prior knowledge matrix (*C*, genes as rows and gene sets as columns). For a given *Y* and *C*, PLIER tries to find loadings for LVs (*Z*, genes as rows and LVs as columns), representation of the original data in latent variable space (*B*, LVs as rows and samples as columns), and an assignment of gene sets to LVs (*U*, gene sets as rows and LVs as columns) by minimizing the following formula:

$$\|Y-ZB\|F2+\lambda_{1}{\left\|Z-CU\right\|}_{F}^{2}+\lambda_{2}\|B\|F2+\lambda_{3}\|U{\|}_{L^{1}},$$

where *λ*_1_, *λ*_2_, and *λ*_3_ are the parameters. The first term represents reconstruction error when converting expression data from gene space to latent variable space. The second term forces latent variables to align with prior knowledge gene sets. The third one is *L*^2^ penalty on *B* to ensure no single LV explained too much. The final term is *L*^1^ penalty on *U* to ensure that a LV is only associated with a few gene sets.

Due to the large size (∼40 GB) of preprocessed Recount3 expression data, we began the PLIER pipeline by precomputing the initialization for PLIER with incremental principal component analysis (PCA) in scikit-learn (Pedregosa et al., 2011). We then used the expression compendium, prior knowledge gene sets, and PCA initializations to train a PLIER model with default parameters. The resulting task took 2 d to run and yielded 196 latent variables.

### RNA-seq processing

RNA-seq reads from male mouse microglia and astrocytes (Pan et al., 2020) were mapped to mm10 reference genome using STAR (v2.7.1a; Dobin et al., 2013) with parameters: –outFilterMismatchNmax 3 –outFilterMultimapNmax 1 –alignSJoverhangMin 8. Gene level read counts were prepared using featureCounts (subread v1.6.1; Liao et al., 2014). The gene annotation file used in featureCounts was downloaded from Recount3 (https://rna.recount.bio/docs/raw-files.html#annotation-files). The gene expression data were TPM normalized and *Z*-scored in the same way as Recount3 training dataset.

### LV significance for mouse aging RNA-seq data

We first transformed the mouse aging expression data from gene space (*Y*_target_) to latent variable space (*B*_target_) using a custom Python script based on this equation: *B*_target_ = (*Z^T^Z* + *λ*_2_*I*)^−1^*Z^T^Y*_target_ (as in Taroni et al., 2019), where *I* is an identity matrix. To determine which latent variables were associated with aging in each disease and cell type (WT microglia, WT astrocyte, AD microglia, and AD astrocyte), we used a linear model. In the model, we look at LV expression as a function of mouse age for each LV by treating development stage (in month) as a numerical variable. To correct the *p* values for multiple testing, we used the Benjamini–Hochberg procedure (FDR; Benjamini and Hochberg, 1995). LVs with FDR < 0.05 were considered to be significantly associated. Overlap of significant LVs was plotted with nVennR (Pérez-Silva et al., 2018). Gene sets associated with each LV were visualized using pheatmap (Kolde, 2019).

### Clustering

We selected the latent variables significantly associated with aging in mouse microglia as a biological starting point. We then used these latent variables to query the training data and see which studies seemed associated with the same biological signals. To do so, we used *k*-means clustering with a *k* of 2, to look for experiments where there was some experimental condition that affected the latent variable. We then ranked the top 10 studies based on their silhouette scores and looked to see which conditions were associated with relevant experimental variables.

### Hardware and software

The PLIER model training was performed on the Penn Medicine high-performance computing cluster running CentOS v7.8. We used R v4.1.0 and PLIER v0.1.6 for the pipeline. The full pipeline takes ∼2 weeks to run, with the main bottlenecks being the Recount3 data download, which takes 1 week to run, and training the PLIER model, which takes 2 d on a compute node (Dell R940 big memory system) with 250 GB of random access memory (RAM). Transforming mouse aging expression on to LV space was performed on Dell C6420 Quad node systems. This step can also be easily accomplished on a personal computer.

### Web server

The Web server for visualizing the results was built on top of the ADAGE (Analysis using Denoising Autoencoders of Gene Expression) Web app framework (Tan et al., 2017). The main changes we made were to substitute the latent variables and gene sets from our trained PLIER model and to forgo uploading the input expression data as the mouse compendium we used was much larger than the input expression for ADAGE.

### Data and code availability

All data and code used in this study can be found at https://github.com/greenelab/mousiplier.

## Results

### MousiPLIER learned latent variables with ideal pathway-level and gene-level sparsity

**Figure 1.**
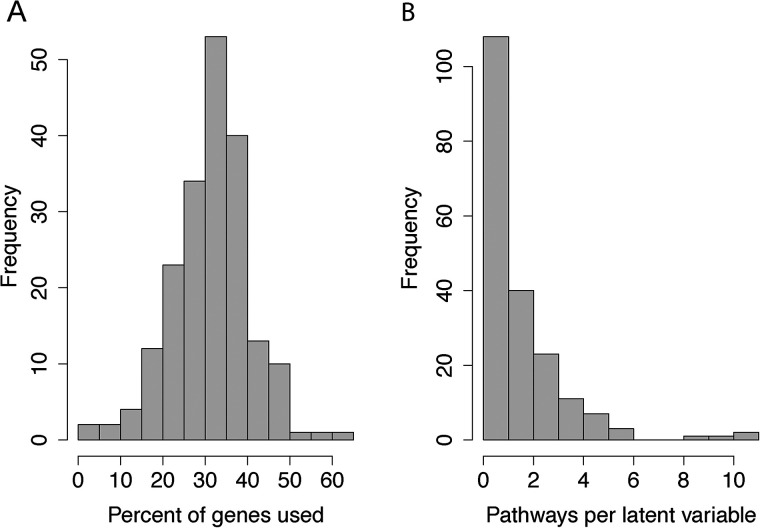
mousiPLIER learned latent variables with ideal pathway-level and gene-level sparsity. ***A***, The distribution of the percentage of genes from the training set used per latent variable. ***B***, The distribution of the number of prior knowledge gene sets used per latent variable.

We trained mousiPLIER using on-disk PCA implementation to initialize PLIER, modified the pipeline to work with mouse data, and used a high-memory compute node to manage the size of the matrix decomposition (see Materials and Methods). The resulting model had 196 latent variables with ideal pathway-level and gene-level sparsity. The per latent variable distribution had an average of 65% sparsity, such that the latent variables tended to use only ∼35% of the genes in the training data (Fig. 1*A*). While many of the latent variables corresponded to no pathways, indicating signals in the training data not passed in as prior knowledge, those that remained corresponded to few pathways (Fig. 1*B*). This optimal pathway-level and gene-level sparsity allowed us to interrogate individual latent variables that corresponded to a small number of biological functions.

### MousiPLIER identified LVs were associated with aging

**Figure 2.**
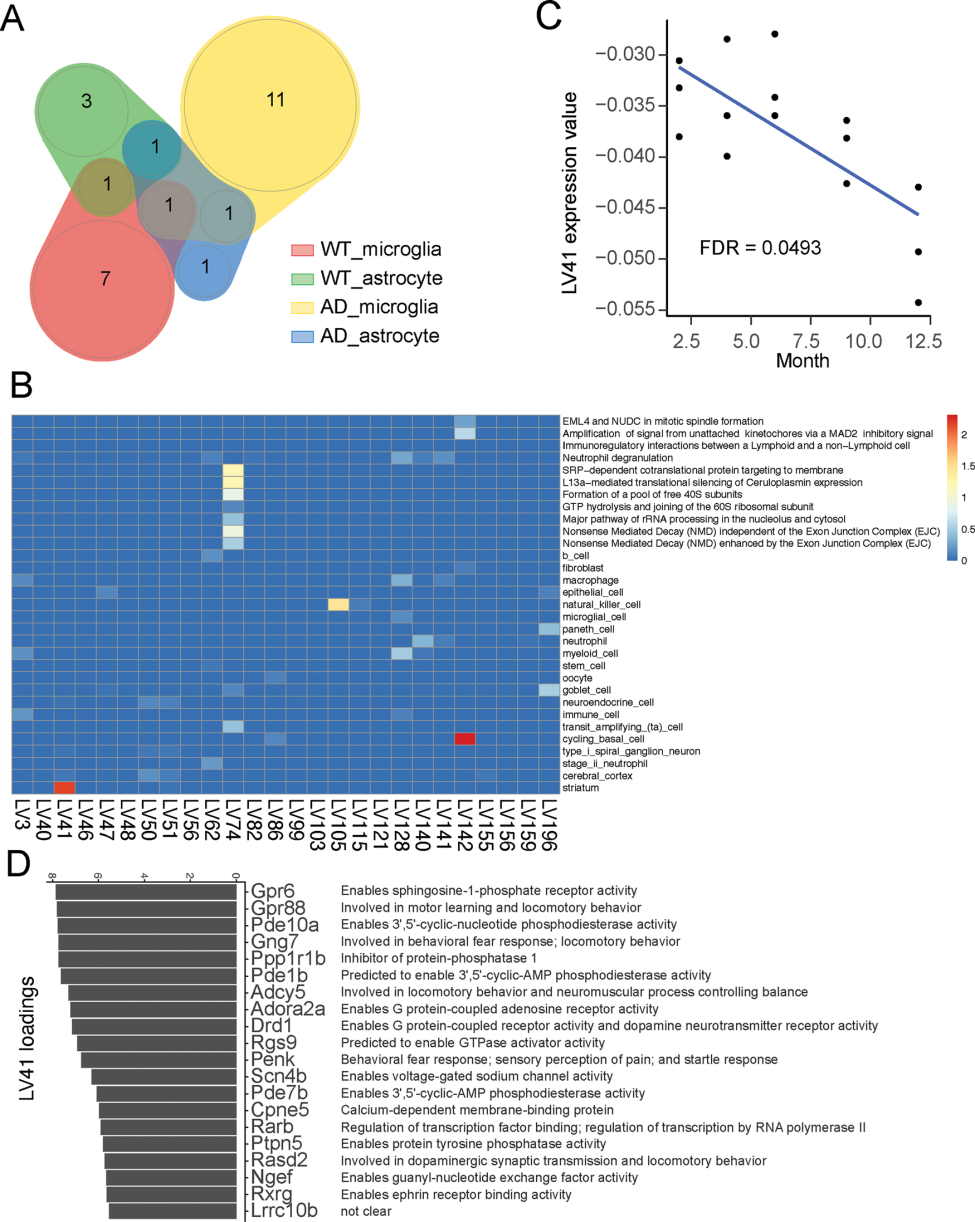
mousiPLIER identified LVs associated with aging. ***A***, Venn diagram showing the number of significant LVs and their overlap across cell types and experimental conditions. ***B***, LV41 is deceased significantly in wild-type microglia during aging. ***C***, A heatmap showing significant LV-associated biological pathways or cell type markers. A linear model is used to test the effect of aging on each LV. *p* values are adjusted for multiple comparisons using Benjamini–Hochberg method. An LV is differentially expressed if FDR < 0.05. LV, latent variable; AD, Alzheimer's disease; WT, wild-type. ***D***, Top 20 genes with highest weight associated with LV41 and their potential annotation. LV, latent variable.

To validate the utility of mousiPLIER, we next interrogated brain-relevant latent variables that our mousiPLIER learned from the training data. To this end, we analyzed an individual study on mouse brain aging (Pan et al., 2020). This study measures wild-type and Alzheimer's disease (APP-PS1) mouse gene expression in microglia and astrocytes at five ages across adulthood. We first projected the RNA-seq data from this study (gene space) to mousiPLIER (LV space). Then, we used a linear model to identify the latent variables that changed significantly across developmental aging. mousiPLIER identified a specific set of significantly changed LVs in each condition in the study (Fig. 2*A*, Table 1). These mousiPLIER-learned LVs are aligned to diverse, prior-knowledge gene sets (Fig. 2*B*). In particular, latent variable 41 uniquely corresponds to striatal signal. This latent variable decreased throughout aging in wild-type microglia. Top-weighted genes of latent variable 41 were functionally associated with striatal cell type specificity (Fig. 2*C*,*D*). Previous studies show that STEP (encoded by *Ptpn5*) and PDE10A (encoded by *Pde10a*) protein levels decline in striatum during aging (Fazio et al., 2017; Cases et al., 2018). The identification of these genes in LV41 indicate that microglia might exhibit molecular processes that occur in aging. As latent variable 41 is mapped to a single gene set and has a potential role in aging, we focus on this latent variable in the rest of the study.

**Table 1.** Significantly changed latent variables in each condition

LV_ID	Genotype	Region	Adjusted *p* value
LV3	WT	Microglia	0.0385
LV40	AD	Astrocyte	0.0408
LV40	AD	Microglia	0.0159
LV41	WT	Microglia	0.0493
LV46	WT	Astrocyte	0.0416
LV46	WT	Microglia	0.0159
LV47	WT	Astrocyte	0.0165
LV48	WT	Microglia	0.0385
LV50	AD	Astrocyte	0.0385
LV51	WT	Microglia	0.0377
LV56	AD	Microglia	0.0165
LV62	AD	Microglia	0.0398
LV74	AD	Microglia	0.0422
LV82	AD	Microglia	0.0061
LV86	WT	Microglia	0.0398
LV99	AD	Astrocyte	0.0385
LV99	AD	Microglia	0.0159
LV99	WT	Microglia	0.0086
LV103	AD	Microglia	0.0385
LV105	AD	Microglia	0.0398
LV115	WT	Microglia	0.0007
LV121	AD	Microglia	0.0398
LV128	AD	Microglia	0.0086
LV140	AD	Microglia	0.0385
LV141	AD	Microglia	0.0214
LV142	WT	Astrocyte	0.0057
LV155	AD	Astrocyte	0.0477
LV155	WT	Astrocyte	0.0165
LV156	WT	Astrocyte	0.0497
LV159	WT	Microglia	0.0165
LV196	AD	Microglia	0.0165

Gene expression data were converted into latent space and tested for differential expression during aging. WT, wild type; AD, Alzheimer disease; LV, latent variable.

### Latent variable 41 demonstrated the biological relevance of mousiPLIER latent variables

Having identified microglia-associated latent variables of interest, we next sought to validate the relevance of this gene set by finding which studies in the mousiPLIER training data responded strongly to them. To do so, we developed a novel method to rank studies based on their latent variable weights. More precisely, we performed *k*-means clustering with a *k* of two on each study in each latent variable space and ranked studies by their silhouette scores, a metric measuring the degree to which clusters are separated from each other. Using this approach, we identified studies that contained samples distinguishable by their values for our latent variables of interest.

**Figure 3.**
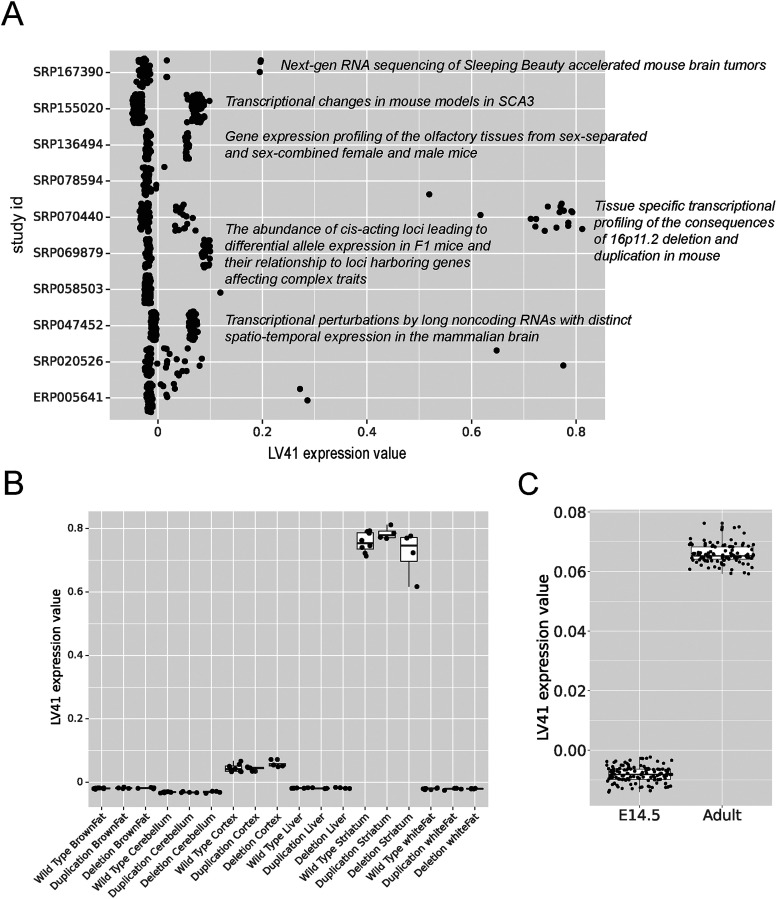
Latent variable 41 demonstrated the biological relevance of mousiPLIER latent variables. ***A***, Studies with the 10 highest silhouette scores after clustering according to LV41 expression values. ***B***, LV41 expression values are higher for striatal tissue than other tissues in SRP070440. ***C***, Effects of development on LV41. Samples are collapsed based on developmental timepoints.

We focused this approach on latent variable 41. We found that many of the studies with the highest silhouette scores for latent variable 41 indicated processes occurring in the brain (Fig. 3*A*). We dug deeper into which specific samples were present in each cluster and found that latent variable 41 was in fact learning something brain (and more specifically striatum) related (Fig. 3).

For example, in a study to delineate tissue-specific transcriptional consequences of copy number variant within 16p11.2, a common cause of autism spectrum disorder, gene expression data were profiled from three genotypes (wild type, deletion, and duplication of the 16p11.2 region) and six tissues (brown fat, liver, white fat, cerebellum, cortex, and striatum; https://www.ncbi.nlm.nih.gov/geo/query/acc.cgi?acc=GSE76872). The LV experimental values in the striatal samples, irrespective of the genotype, clearly stand apart from the other tissues (Fig. 3*B*). Additionally, in a study to investigate transcriptional effects of selected long noncoding RNAs (lncRNAs), mRNA expression is generated from embryonic and adult whole brains of wild-type and lncRNA knock-out mouse (Goff et al., 2015). LV41 expression is higher in adult (7.6–14.1 weeks) samples compared with embryonic day 14.5 timepoint regardless of knock-out status (Fig. 3*C*), supporting the association between latent variable 41 and aging found in the study (Pan et al., 2020) we used to derive the latent variables.

### Web server

**Figure 4.**
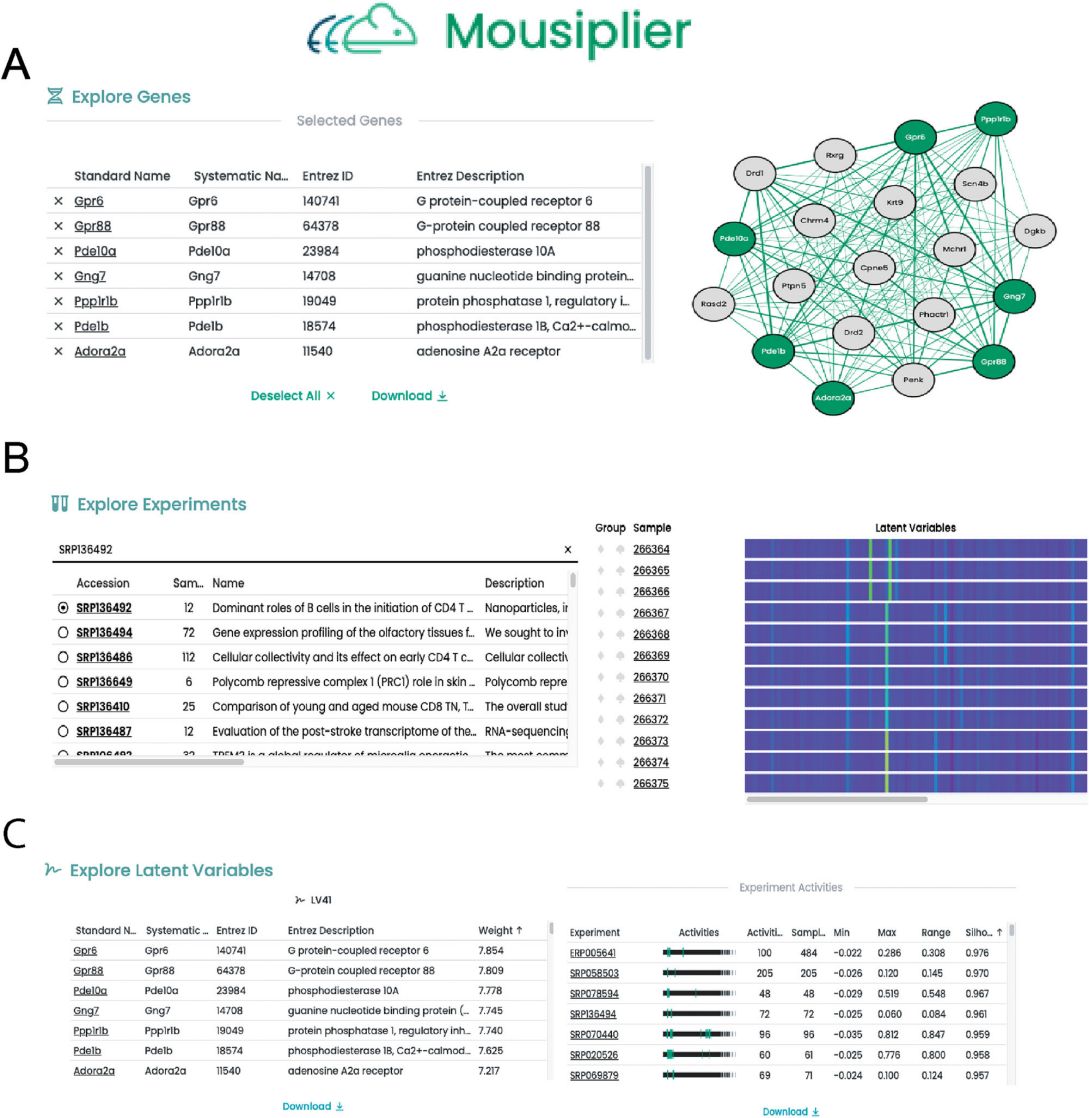
Snapshot of mousiPLIER Web server showing its functions. ***A***, An example to visualize the gene network of LV41 associated genes. ***B***, An example to explore experiments and view samples’ activities in mousiPLIER latent space. ***C***, An example to explore latent variables. Gene weights and the clustering of experiments for each LV is displayed and can be easily downloaded.

To allow others to independently examine mousiPLIER learned latent variables, we developed a Web server at http://mousiplier.greenelab.com/. This server allows users to list the genes present in, visualize which experiments had high cluster scores for, and see which biological pathways participate in each latent variable (Fig. 4).

## Discussion

In this paper we developed mousiPLIER and established proof of concept for training extremely large PLIER models on mouse data. The learned latent variables mapped to various biological processes and cell types. Further, we applied a novel approach for surfacing latent-variable relevant experiments from an expression compendium. Specifically, we clustered training experiments based on latent variable values, allowing us to query a large compendium for experiments pertaining to mouse striatal aging. Finally, we created a Web server to make the model's results more easily accessible to other scientists.

Although we focused our analysis on LV41 to validate the utility of mousiPLIER, we identified other significantly changed LVs that are associated with aging-relevant pathways. For example, LV142 is associated with cycling basal cells (Fig. 2*B*) and is significantly decreased in WT astrocytes. The top weighted genes of LV142 contain cell division genes, such as Cytoskeleton-associated protein 2-like (Ckap2l) and Nucleolar and spindle-associated protein 1 (Nusap1). Association of such cell cycle markers in basal cells is consistent with their high turnover rate. The observation that WT astrocytes exhibit decreased LV142 in aged mice is likely due to reduced local proliferation of astrocytes during aging, as shown in mouse dentate gyrus (Schneider et al., 2022). In addition, LV74 is significantly increased in AD microglia. Top weighted genes in LV74 encode ribosomal proteins, and LV74 is associated with several pathways related to mRNA translation and protein translocation (Fig. 2*B*). One of the associated pathways is signal recognition particle (SRP)–dependent cotranslational protein targeting to membrane, which is the top enriched pathway for differentially expressed genes between AD microglia and healthy microglia (Wang and Li, 2021). More specifically, increased microglial expression of genes in this pathway is associated with more severe AD pathology (Patel et al., 2020). This is consistent with our result that the expression of LV74 is gradually increased in AD microglia during aging, highlighting the importance of this pathway in AD progression. Further study of these LVs may provide clues to biological pathways relevant to aging that are utilized for different functions across distinct cell types.

Of note, LV115 and LV105 are significantly increased in WT microglia and AD microglia, respectively. This latent variable is associated with the natural kill (NK) cell marker (Fig. 2*B*). We suspect that the isolated microglia contain a small portion of NK cells as CD45^low-to-intermediate^ and CD11b (markers used in Pan et al., 2020) are also present in NK cells (Chiossone et al., 2009; He et al., 2016). Moreover, the expression of CD45 from microglia can be upregulated in aged brain (Honarpisheh et al., 2020), reducing the specificity of CD45 expression to select microglia during aging. Our speculation is further supported by the dramatic increase of NK cells in aged mouse brain (18 months old) compared with 3-month-old mice (Jin et al., 2021). Finally, high NK cell number, though lower than wild type, is also observed in 7–8 months 3xTa-AD mouse brain (Y. Zhang et al., 2020), consistent with the increase of NK signal during aging.

Traditional pathway analyses are a powerful tool to gain biological insights from RNA-seq data. However, there are limitations for such methods. First, for pathway analysis tools using a subset of differentially expressed genes (DEGs), there is no standard cutoff for selecting DEGs (Khatri et al., 2012). Second, examining ∼20,000 expressed genes in mouse, for example, imposes a high multiple testing burden. Finally, although a ranked gene list could be theoretically applied provided to existing pathway analysis tools, these packages cannot distinguish true pathway signal from batch effects due noise generated by the technical variability common in RNA-sequencing (Leek et al., 2010). mousiPLIER overcomes those issues by transforming gene expression data into latent variable space. The resulting space only has 196 dimensions, greatly reducing multiple testing burden. Moreover, mousiPLIER reduces technical noises by separating technical noises into pathway-irrelevant LVs, which is extremely valuable in comparing transcriptomic data from different studies or laboratories. Researchers can test decreased or increased pathway-associated LVs directly at the LV space. After finding LVs significantly changed among specific experimental conditions, users can interrogate the training dataset to see if the LVs demonstrate biological relevance as we did for the latent variable 41 in the manuscript. Users may further pick genes based on the loadings of the LVs for experimental examination.

Although mousiPLIER is trained on a compendium of bulk RNA-seq data, it could be potentially applied to single-cell transcriptomic datasets. The input for mousiPLIER is a read count table where rows are genes and columns are samples. But the result of a typical single-cell RNA-seq data is a table with columns being sequenced cells, and single-cell level count is too sparse to be used by mousiPLIER directly. Users can aggregate single-cell level count into sample level count for a specific cell type and then apply the pseudobulk samples to mousiPLIER.

While we successfully transformed a study from outside the training data into the latent space and identified study-specific latent variables, application of mousiPLIER was not universally successful across transcriptomic studies, such as RNA-seq data from drug addiction (Carpenter et al., 2020). This may be due to high variance across samples in training compendium, too few samples in the study of interest to generate sufficient statistical power, or other factors. In these cases, there is not yet a method to select meaningful latent variables to guide downstream analyses.

Finally, as a linear model, PLIER can only approximate nonlinear relationships between the genes used to train the model and the learned biological pathways. While we do not expect this to have a large impact (Heil et al., 2023), incorporating prior knowledge into nonlinear models such as neural networks is an exciting field of research and a potential improvement for the MultiPLIER and mousiPLIER frameworks. Going forward, our model and Web server will allow scientists to explore the latent space of their own experiments and learn about relevant biological pathways and cell types.

## Synthesis

Reviewing Editor: Sam Golden, The University of Washington Decisions are customarily a result of the Reviewing Editor and the peer reviewers coming together and discussing their recommendations until a consensus is reached. When revisions are invited, a fact-based synthesis statement explaining their decision and outlining what is needed to prepare a revision will be listed below. The following reviewer(s) agreed to reveal their identity: Michelle Jin. Note: If this manuscript was transferred from JNeurosci and a decision was made to accept the manuscript without peer review, a brief statement to this effect will instead be what is listed below.

Dear Drs. Greene and Heller,

Overall, the reviewers of your manuscript provided highly congruent comments that will strengthen the manuscript with relatively straightforward additions. They were both supportive of the manuscript and related package, although the github link currently retrieves a 404 error page making it impossible to assess the actual code. Please have this corrected prior to revisions being submitted.

Please provide point-by-point rebuttals to the reviewer comments. Both reviewers had critical comments relating to better understanding the relationships between latent variables and experimental conditions and data interpretation.

REVIEWER 1:

In this manuscript, Zhang et al. applied the pathway-level information extractor (PLIER) approach, an unsupervised machine learning framework which automatically identifies significant biological pathways from gene expression data, to an unprecedented large training compendium of gene expression in mice (mousiPLIER). To validate the latent variables identified as biologically relevant gene sets, they transferred knowledge of latent variables to a smaller independent gene expression dataset derived from astrocytes and microglia of wild-type and Alzheimer's disease model mice of varying ages (Pan et al., 2020). They identified one latent variable (LV41) that was significantly associated with aging in wild-type microglia, and which associated with an expression signal characteristic of striatal cells. They confirm this striatal association by applying a custom approach to investigate which studies in the training compendium were most contributory to the LV41 signal. They additionally reanalyzed one of these studies with regards to aging and confirmed an association between aging and the LV41 gene expression signal. Lastly, the authors have made the output of mousiPLIER public for open exploration on a web server. Overall, the paper would be improved after addressment of the major and minor concerns listed below along with a significant expansion of the discussion section. Given that the mousiPLIER results would serve as a significant community resource to those performing mouse gene expression analyses, I believe this article would be of interest to the readers of this journal pending these major revisions.

Major:

1) Fig 1B. shows that a large proportion of latent variables seem to correspond to one or a few pathways already annotated or previously compiled in the training dataset. This begs the question of how useful mousPLIER approach of examining latent variables is compared to traditional pathway analyses of gene expression in mice. Does this mapping of latent variables better encapsulate interactions between pathways? A comparison with traditional pathway analysis or at the very least an expanded discussion is missing.

2) There is insufficient documentation of the methods used to transform gene expression on to latent variable space. Although this may be included in the attached GitHub link for the code (this link did not work for me), the mathematical approach should be referenced or outlined explicitly in the main text.

3) For the linear model analysis, details regarding this analysis are missing in the text. For example, were disease and cell type from the expression dataset from Pan et al., 2020 treated as separate factors?

4) Reporting of Fig. 2C is missing mention of the other latent variables associated with aging in WT microglia. For example, there is also a strong association in LV42 pertaining to cycling basal cells, but there is no mention of this at all in the results or discussion. While basal cells are in the epidermis, their high turnover rate may imply a shared gene regulation pathway across WT microglia and basal cells during the aging process. Additionally, there are associations with LV74, and LV105. This point should be addressed in the discussion. Are these other latent variables biologically relevant and do they make sense in the context of aging?

5) "To narrow down the scope of the analysis, we also validated the biological relevance of the latent variables associated with wild-type microglial cells."

a) There are insufficient details of how this validation was done, making this statement ambiguous.

b) This justification for only focusing on wild-type (WT) microglial cells for the analysis feels insufficient. Why was this not also performed for AD microglial cells, as this would add an interesting translational significance to the study.

c) Fig. 2A indicates there is one aging-associated latent variable which overlaps between WT and AD microglia. It appears like an oversight not to look into the biological significance of this latent variable that shows "conserved" changes with aging, even in a diseased state.

6) Figure 3C is confusing. The text mentions a study examining gene expression in wild-type or lncRNA knockout mice at either embryonic or adult ages. This is four groups, but the graph shows two. Is this association of LV41 regardless of knockout status, and thus collapsed, across this condition? If so, this should be explicitly written rather than assumed.

7) An expanded discussion conjecturing why LV 41 emerges as a striatal and aging related signature from WT microglia gene expression data is missing. To my knowledge, the Pan et al. 2020 study did not specifically collect microglia samples from mouse striatum.

Minor:

1) Fixing of minor grammatical errors in the manuscript would improve readability.

2) Some abbreviations that are not explained during their first use (e.g. RPKM).

3) The validation of mouseiPLIER is not on "neuronal gene regulation" as stated on page 5 but on microglia and astrocytes.

4) Font sizes could be increased for:

a. Fig. 2A. (the number of latent variable numbers)

b. Fig. 3C

5) It makes more logical sense that Fig. 2C should come before Fig. 2B.

6) There is insufficient description of the hardware and software used for MousePlier.

7) Figure 2D. Without prior knowledge of these genes, it is difficult to make sense of this figure. It would be helpful to include some annotation or instead represent this figure as a table with annotations.

8) "Because we were interested in mouse brains in particular, we rounded out our set of prior information by manually selecting marker genes for the striatum, midbrain, and cerebellum."

a. More justification needs to be included on why marker genes were only selected for the striatum, midbrain and cerebellum? Why not cortical genes and why would the marker genes selected be more suitable for the specific investigation of mouse brains?

REVIEWER 2:

In this manuscript by Zhang and colleagues, the team introduces "MousiPLIER," a machine learning model developed to extract insights from gene expression datasets. The model was trained on an extensive set of publicly available mouse brain gene expression data, enabling it to identify 'latent variables' or underlying patterns in the data.

These latent variables have been utilized to detect changes in gene expression across different developmental stages of the mouse brain. To facilitate broader access to MousiPLIER, the authors have integrated it into a web-based platform, allowing researchers to explore mouse brain gene expression datasets.

Overall, the paper is easy to follow and is a solid advancement for studying bulk gene expression in the mouse brain. The paper provides a structured overview of the process and outcomes of analyzing bulk gene expression in the mouse brain using MousiPLIER.

A few points of consideration for the authors might include:

1. While MousiPLIER is currently designed for bulk transcriptomic data, there could be potential in exploring its adaptability to single-cell or single-nuclei transcriptomic datasets.

2. It might be beneficial to discuss methods or approaches for validating the relationships that the model identifies between latent variables and specific experimental conditions.

## Author Response

Overall, the reviewers of your manuscript provided highly congruent comments that will strengthen the manuscript with relatively straightforward additions. They were both supportive of the manuscript and related package, although the github link currently retrieves a 404 error page making it impossible to assess the actual code. Please have this corrected prior to revisions being submitted.

We have corrected the GitHub link setting to public access.

Please provide point-by-point rebuttals to the reviewer comments. Both reviewers had critical comments relating to better understanding the relationships between latent variables and experimental conditions and data interpretation.

We thank reviewers for their thoughtful comments, which greatly improved the manuscript.

We have addressed each individual comment as detailed below and in the revised manuscript.

Reviewers' comments are in black, and our responses are in blue.

REVIEWER 1:

In this manuscript, Zhang et al. applied the pathway-level information extractor (PLIER) approach, an unsupervised machine learning framework which automatically identifies significant biological pathways from gene expression data, to an unprecedented large training compendium of gene expression in mice (mousiPLIER). To validate the latent variables identified as biologically relevant gene sets, they transferred knowledge of latent variables to a smaller independent gene expression dataset derived from astrocytes and microglia of wild-type and Alzheimer's disease model mice of varying ages (Pan et al., 2020). They identified one latent variable (LV41) that was significantly associated with aging in wild-type microglia, and which associated with an expression signal characteristic of striatal cells. They confirm this striatal association by applying a custom approach to investigate which studies in the training compendium were most contributory to the LV41 signal. They additionally reanalyzed one of these studies with regards to aging and confirmed an association between aging and the LV41 gene expression signal. Lastly, the authors have made the output of mousiPLIER public for open exploration on a web server. Overall, the paper would be improved after addressment of the major and minor concerns listed below along with a significant expansion of the discussion section. Given that the mousiPLIER results would serve as a significant community resource to those performing mouse gene expression analyses, I believe this article would be of interest to the readers of this journal pending these major revisions.

Major:

1) Fig 1B. shows that a large proportion of latent variables seem to correspond to one or a few pathways already annotated or previously compiled in the training dataset. This begs the question of how useful mousPLIER approach of examining latent variables is compared to traditional pathway analyses of gene expression in mice. Does this mapping of latent variables better encapsulate interactions between pathways? A comparison with traditional pathway analysis or at the very least an expanded discussion is missing.

Thank you for this comment. The result that a large proportion of latent variables correspond to one or a few pathways is what PLIER tries to accomplish (Mao et al., 2019). We added the following to compare traditional pathway analyses and mousiPLIER (line 264-276): "Traditional pathway analyses are a powerful tool to gain biological insights from RNA-seq data.

However, there are limitations for such methods. First, for pathway analysis tools using a subset of differentially expressed genes (DEGs), there is no standard cutoff for selecting DEGs (Khatri et al., 2012). Second, examining ~20,000 expressed genes in mouse, for example, imposes a high multiple testing burden. Finally, although a ranked gene list could be theoretically applied to existing pathway analysis tools, these packages cannot distinguish true pathway signal from batch effects due noise generated by the technical variability common in RNA-sequencing (Leek et al., 2010). mousiPLIER overcomes those issues by transforming gene expression data into latent variable space. The resulting space only has 196 dimensions, greatly reducing multiple testing burden. Moreover, mousiPLIER reduces technical noise by designating it as pathway irrelevant LVs, which is extremely valuable in comparing transcriptomic data from different studies or laboratories. Researchers can then test decreased or increased pathway-associated LVs directly at the LV space." 2) There is insufficient documentation of the methods used to transform gene expression on to latent variable space. Although this may be included in the attached GitHub link for the code (this link did not work for me), the mathematical approach should be referenced or outlined explicitly in the main text.

Thank you for pointing this out. We have corrected the GitHub link setting to public access. In addition, we added the following description about how gene expression data are transformed on to latent variable space and explicitly referenced the original PLIER paper in the main text.

Please see PLIER in the Materials and Methods for the details (line 101-113 and line 128 - 130).

We transformed training dataset on to latent space by minimizing: where Y is raw gene expression matrix, C is prior knowledge gene set matrix, Z is loading matrix, B is the expression data in latent space, U is a matrix indicating association between latent variable and gene sets.

We first transformed the mouse aging expression data from gene space (Ytarget) to latent variable space (Btarget) using a custom Python script based on this equation: Btarget = (Z TZ + λ2I) - 1Z TYtarget (as in Taroni et al., 2019), where I is an identity matrix.

3) For the linear model analysis, details regarding this analysis are missing in the text. For example, were disease and cell type from the expression dataset from Pan et al., 2020 treated as separate factors? We don't add disease and cell type in the linear model. Instead, for each condition (wild-type microglia, wild-type astrocyte, Alzheimer's disease microglia, and Alzheimer's disease astrocyte), we look at LV expression as a function of mouse age (in months) for each LV. We have added this information to the revised manuscript (line 130-133).

4) Reporting of Fig. 2C is missing mention of the other latent variables associated with aging in WT microglia. For example, there is also a strong association in LV42 pertaining to cycling basal cells, but there is no mention of this at all in the results or discussion. While basal cells are in the epidermis, their high turnover rate may imply a shared gene regulation pathway across WT microglia and basal cells during the aging process. Additionally, there are associations with LV74, and LV105. This point should be addressed in the discussion. Are these other latent variables biologically relevant and do they make sense in the context of aging? We are grateful for the recommendation to include additional information in the revision. We have added Table 1 to show all LVs that are significantly changed in each condition.

Note that we think that this comment refers to LV142, which is significantly changed in WT astrocytes instead of WT microglia (the original Fig. 2C/revised Fig. 2B does not include any LV42).

These LVs are discussed (line 233-262):

Although LV41 was focused to validate the utility of mousiPLIER, we identified other significantly changed LVs that are associated with aging-relevant pathways. For example, LV142 is associated with cycling basal cell (Fig. 2B) and is significantly decreased in WT astrocytes. The top weighted genes of LV142 contain cell division genes, such as Cytoskeleton-associated protein 2-like (Ckap2l) and Nucleolar and spindle-associated protein 1 (Nusap1). That basal cells have these cell cycle markers is consistent with basal cells' high turnover rate. The observation that WT astrocytes exhibit decreasing in LV142 in aged mouse is likely due to reduced local proliferation activity in astrocytes during aging, which has been shown in mouse dentate gyrus (Schneider et al., 2022). In addition, LV74 is significantly increased in AD microglia. Top weighted genes in LV74 encode ribosomal proteins, and LV74 are associated with several pathways related to mRNA translation and protein translocation (Fig. 2B). One of the associated pathways is signal recognition particle (SRP)-dependent cotranslational protein targeting to membrane, which is the top enriched pathway for differentially expressed genes between AD microglia and healthy microglia (Wang and Li, 2021). More specifically, increased microglial expression of genes in this pathway is associated with more severe AD pathology (Patel et al., 2020). This is consistent with our result that the expression LV74 is gradually increased in AD microglia during aging, highlighting the importance of this pathway in AD progression. Further study of these LVs may provide clues to biological pathways relevant to aging that are utilized for different functions across distinct cell types.

Of note, LV115 and LV105 is significantly increased in WT microglia and AD microglia, respectively. This latent variable is associated with the natural kill (NK) cell marker (Fig. 2B). We suspect that the isolated microglia contain a small portion of NK cells as CD45low-to-intermediate and CD11b (markers used in Pan et al. 2020) are also present in NK cells (Chiossone et al., 2009;

He et al., 2016). Moreover, the expression of CD45 from microglia can be upregulated in aged brain (Honarpisheh et al., 2020), reducing the specificity of using CD45 expression level to select microglia during aging. Our speculation is further supported by the dramatic increase of NK cells in aged mouse brain (18 months old) compared to 3-month-old mice (Jin et al., 2021).

Finally, high NK cell number, though lower than wild type, is also observed in 7-8 months 3xTa-AD mouse brain (Zhang et al., 2020), consistent with the increase of NK signal during aging.

5) "To narrow down the scope of the analysis, we also validated the biological relevance of the latent variables associated with wild-type microglial cells." a) There are insufficient details of how this validation was done, making this statement ambiguous.

We validated the biological relevance of the latent variable by examining the training dataset, which is the next section of the paper. We remove this sentence to avoid confusion (line 190- 191). b) This justification for only focusing on wild-type (WT) microglial cells for the analysis feels insufficient. Why was this not also performed for AD microglial cells, as this would add an interesting translational significance to the study.

This comment, and that in question 4, strengthen the manuscript by adding translational significance to the study. The revised manuscript now includes the LVs changed in AD microglia. We found the pathway, (SRP)-dependent cotranslational protein targeting to membrane, is of particular interest for further study (line 233-251).

We initially focused only on WT microglial cells because LV41 is significantly changed in those cells. LV41 could then serve as a test LV to capture striatal signal, i.e., the biological relevance of mousiPLIER-learned latent variable. c) Fig. 2A indicates there is one aging-associated latent variable which overlaps between WT and AD microglia. It appears like an oversight not to look into the biological significance of this latent variable that shows "conserved" changes with aging, even in a diseased state.

We noticed that the overlapped latent variable between WT and AD microglia is LV99 (Table 1).

But LV99 is not associated with any prior gene sets (Fig. 2B). So, LV99 likely corresponds to technical variation or biological signal not captured in the gene sets provided to mousiPLIER while training (Mao et al., 2019).

6) Figure 3C is confusing. The text mentions a study examining gene expression in wild-type or lncRNA knockout mice at either embryonic or adult ages. This is four groups, but the graph shows two. Is this association of LV41 regardless of knockout status, and thus collapsed, across this condition? If so, this should be explicitly written rather than assumed.

Yes, the association is regardless of knockout status and collapsed. We added this information to the main text, figure legend (line 216-218 and line 440-441).

7) An expanded discussion conjecturing why LV 41 emerges as a striatal and aging related signature from WT microglia gene expression data is missing. To my knowledge, the Pan et al.

2020 study did not specifically collect microglia samples from mouse striatum.

Indeed, Pan et al. 2020 collected microglia and astrocytes from whole brain rather than striatum.

The outcome of our study is that the association of LV41 with striatal signal is learned from the training dataset. There are studies showing that STEP protein (encoded by Ptpn5) and PDE10A (encoded by Pde10a) are declined during aging in striatum (Cases et al., 2018)(Fazio et al., 2017). Microglia might show a similar aging process (line 183-190).

Minor:

1) Fixing of minor grammatical errors in the manuscript would improve readability.

We fixed grammatical errors by proofreading the manuscript multiple times (line 124, line 181).

2) Some abbreviations that are not explained during their first use (e.g. RPKM).

We noticed that we used TPM for normalization, instead of RPKM. We fixed the abbreviation issue by adding complete forms for TPM (line 85), PCA (line 115-116), RAM (line 150), and ADAGE (line 154-155).

3) The validation of mouseiPLIER is not on "neuronal gene regulation" as stated on page 5 but on microglia and astrocytes.

Thanks for pointing this out. We removed "neuronal gene regulation".

4) Font sizes could be increased for: a. Fig. 2A. (the number of latent variable numbers) b. Fig. 3C We increased font size for Fig.2A and Fig. 3C.

5) It makes more logical sense that Fig. 2C should come before Fig. 2B.

We swapped Fig. 2C and Fig. 2B.

6) There is insufficient description of the hardware and software used for MousePlier.

We added descriptions of hardware and software used for MousiPLIER (line 145-152). In addition, more details are available from the mousiPLIER github: https://github.com/greenelab/mousiplier/.

7) Figure 2D. Without prior knowledge of these genes, it is difficult to make sense of this figure.

It would be helpful to include some annotation or instead represent this figure as a table with annotations.

We added a brief annotation for those genes in Fig. 2D.

8) "Because we were interested in mouse brains in particular, we rounded out our set of prior information by manually selecting marker genes for the striatum, midbrain, and cerebellum." a. More justification needs to be included on why marker genes were only selected for the striatum, midbrain and cerebellum? Why not cortical genes and why would the marker genes selected be more suitable for the specific investigation of mouse brains? The focus of the Heller Lab is gene regulatory mechanisms that underlie drug addiction (line 289). We intend to expand studies using mousiPLIER to investigate cocaine effects on transcriptome in prefrontal cortex, nucleus accumbens (part of ventral striatum), and ventral tegmental area (located in the midbrain). As such, we focused on these regions.

We have corrected a typo in the original main text (line 98), that refered to cerebellum marker. and is now corrected to cortical genes as shown in Fig. 2B. In the discussion we describe our findings, such that we did not find significantly changed LVs upon cocaine treatment. We conclude that projecting data into latent space is not universally successful due to variability across samples in training dataset or lack of statistical power (line 287-292).

REVIEWER 2:

In this manuscript by Zhang and colleagues, the team introduces "MousiPLIER," a machine learning model developed to extract insights from gene expression datasets. The model was trained on an extensive set of publicly available mouse brain gene expression data, enabling it to identify 'latent variables' or underlying patterns in the data.

These latent variables have been utilized to detect changes in gene expression across different developmental stages of the mouse brain. To facilitate broader access to MousiPLIER, the authors have integrated it into a web-based platform, allowing researchers to explore mouse brain gene expression datasets.

Overall, the paper is easy to follow and is a solid advancement for studying bulk gene expression in the mouse brain. The paper provides a structured overview of the process and outcomes of analyzing bulk gene expression in the mouse brain using MousiPLIER.

A few points of consideration for the authors might include:

1. While MousiPLIER is currently designed for bulk transcriptomic data, there could be potential in exploring its adaptability to single-cell or single-nuclei transcriptomic datasets.

Thank you for the comment. We agree that mousiPLIER is designed for bulk RNA-seq data. We discussed the potential application of mousiPLIER to single-cell RNA-seq in the discussion.

Basically, users can aggregate single-cell level count into sample level count, and then apply mousiPLIER (line 281-286).

2. It might be beneficial to discuss methods or approaches for validating the relationships that the model identifies between latent variables and specific experimental conditions.

Thank you for the comment. After finding LVs significantly changed among specific experimental conditions, users can interrogate the training dataset to see if the LVs demonstrate biological relevance as we did for the latent variable 41 in the manuscript. Users may further pick genes based on the loadings of the LVs for experimental examination (line 276-280).

References for Response to Reviewers Cases S, Saavedra A, Tyebji S, Giralt A, Alberch J, Pérez-Navarro E (2018) Age-related changes in STriatal-Enriched protein tyrosine Phosphatase levels: Regulation by BDNF. Molecular and Cellular Neuroscience 86:41-49.

Chiossone L, Chaix J, Fuseri N, Roth C, Vivier E, Walzer T (2009) Maturation of mouse NK cells is a 4-stage developmental program. Blood, The Journal of the American Society of Hematology 113:5488-5496.

Fazio P, Schain M, Mrzljak L, Amini N, Nag S, Al-Tawil N, Fitzer-Attas CJ, Bronzova J, Landwehrmeyer B, Sampaio C, others (2017) Patterns of age related changes for phosphodiesterase type-10A in comparison with dopamine D2/3 receptors and sub-cortical volumes in the human basal ganglia: A PET study with 18F-MNI-659 and 11C-raclopride with correction for partial volume effect. Neuroimage 152:330-339.

He H, Geng T, Chen P, Wang M, Hu J, Kang L, Song W, Tang H (2016) NK cells promote neutrophil recruitment in the brain during sepsis-induced neuroinflammation. Sci Rep 6:27711.

Honarpisheh Pedram, Lee J, Banerjee A, Blasco-Conesa MP, Honarpisheh Parisa, d'Aigle J, Mamun AA, Ritzel RM, Chauhan A, Ganesh BP, others (2020) Potential caveats of putative microglia-specific markers for assessment of age-related cerebrovascular neuroinflammation. J Neuroinflammation 17:1-13.

Jin W-N, Shi K, He W, Sun J-H, Van Kaer L, Shi F-D, Liu Q (2021) Neuroblast senescence in the aged brain augments natural killer cell cytotoxicity leading to impaired neurogenesis and cognition. Nat Neurosci 24:61-73.

Khatri P, Sirota M, Butte AJ (2012) Ten years of pathway analysis: current approaches and outstanding challenges. PLoS Comput Biol 8:e1002375.

Leek JT, Scharpf RB, Bravo HC, Simcha D, Langmead B, Johnson WE, Geman D, Baggerly K, Irizarry RA (2010) Tackling the widespread and critical impact of batch effects in high-throughput data. Nat Rev Genet 11:733-739.

Mao W, Zaslavsky E, Hartmann BM, Sealfon SC, Chikina M (2019) Pathway-level information extractor (PLIER) for gene expression data. Nat Methods 16:607-610.

Patel S, Howard D, Man A, Schwartz D, Jee J, Felsky D, Pausova Z, Paus T, French L (2020) Donor-Specific transcriptomic analysis of Alzheimer's Disease-Associated hypometabolism highlights a unique donor, ribosomal proteins and microglia. eNeuro 7.

Schneider J, Weigel J, Wittmann M-T, Svehla P, Ehrt S, Zheng F, Elmzzahi T, Karpf J, Paniagua-Herranz L, Basak O, others (2022) Astrogenesis in the murine dentate gyrus is a life-long and dynamic process. EMBO J 41:e110409.

Wang X-L, Li L (2021) Cell type-specific potential pathogenic genes and functional pathways in Alzheimer's Disease. BMC Neurol 21:1-18.

Zhang Y, Fung ITH, Sankar P, Chen X, Robison LS, Ye L, D'Souza SS, Salinero AE, Kuentzel ML, Chittur S V, others (2020) Depletion of NK cells improves cognitive function in the Alzheimer disease mouse model. The Journal of Immunology 205:502-510.
